# Medical Society of the State of Pennsylvania

**Published:** 1850-04

**Authors:** Henry S. Patterson, George B. Kerfoot

**Affiliations:** 92 Arch street, Philadelphia, Recording Secretary; Lancaster, Recording Secretary


MEDICAL SOCIETY OF THE STATE OF PENNSYLVANIA.

The annual meeting of the Society for 1850, will be held in the Controller's Chamber, Athenæum building, Sixth street below Walnut, in the city of Philadelphia, on Wednesday, April 17th, at 11 o'clock, A. M. Delegates from country societies are requested to report as early as possible to the undersigned.

HENRY S. PATTERSON, 92 Arch street, Philadelphia,
GEORGE B. KERFOOT, Lancaster,
Recording Secretaries.

(We hope the attendance will be large. Our friends from all parts of the State may rest assured that a cordial welcome awaits them.)

---

It is announced by the Boston Medical and Surgical Journal, that Drs. Forbes and Marshall Hall are about to visit this country.

